# Prognostic significance and function of the vacuolar H^+^-ATPase subunit V1E1 in esophageal squamous cell carcinoma

**DOI:** 10.18632/oncotarget.10340

**Published:** 2016-06-30

**Authors:** Sung Wook Son, Seok-Hyung Kim, Eun-Yi Moon, Dong-Hoon Kim, Suhkneung Pyo, Sung Hee Um

**Affiliations:** ^1^ Department of Molecular Cell Biology, Samsung Biomedical Research Institute, Sungkyunkwan University School of Medicine, Gyeonggi-do, 16419, Korea; ^2^ Department of Pathology, Samsung Medical Center, Sungkyunkwan University School of Medicine, Seoul, 06351, Korea; ^3^ Department of Health Sciences and Technology, Samsung Advanced Institute for Health Sciences and Technology, Samsung Medical Center, Sungkyunkwan University, Seoul, 06351, Korea; ^4^ Department of Bioscience and Biotechnology, Sejong University, Seoul, 05006, Korea; ^5^ Department of Pharmacology, Korea University College of Medicine, Seoul, 02841, Korea; ^6^ Division of Immunopharmacology, School of Pharmacy, Sungkyunkwan University, Gyeonggi-do, 16419, Korea

**Keywords:** V-ATPase subunit V1E1, esophageal squamous cell carcinoma (ESCC), cell motility, aerobic glycolysis, prognosis

## Abstract

Vacuolar H^+^-ATPase (V-ATPase), a hetero-multimeric ATP-driven proton pump has recently emerged as a critical regulator of mTOR-induced amino acid sensing for cell growth. Although dysregulated activity of cell growth regulators is often associated with cancer, the prognostic significance and metabolic roles of V-ATPase in esophageal cancer progression remain unclear. Here, we show that high levels of V-ATPase subunit V1E1 (V-ATPase V1E1) were significantly associated with shortened disease-free survival in patients with esophageal squamous cell carcinoma (ESCC). Multivariate analysis identified the V-ATPase V1E1 as an independent adverse prognostic factor (hazard ratio;1.748, *P* = 0.018). In addition, depletion of V-ATPase V1E1 resulted in reduced cell motility, decreased glucose uptake, diminished levels of lactate, and decreased ATP production, as well as inhibition of glycolytic enzyme expression in TE8 esophageal cancer cells. Consistent with these results, the Cancer Genome Atlas (TCGA) data and Gene Set Enrichment Analysis (GSEA) showed a high frequency of copy number alterations of the V-ATPase V1E1 gene, and identified a correlation between levels of V-ATPase V1E1 mRNA and Pyruvate Kinase M2 (PKM2) in ESCC. High expression levels of both V-ATPase V1E1 and phosphorylated PKM2 (p-PKM2), a key player in cancer metabolism, were associated with poorer prognosis in ESCC. Collectively, our findings suggest that expression of the V-ATPase V1E1 has prognostic significance in ESCC, and is closely linked to migration, invasion, and aerobic glycolysis in esophageal cancer cells.

## INTRODUCTION

ESCC is an aggressive malignant tumor with a high mortality rate [1]. The 5-year survival rate of ESCC patients after surgery is 30% to 45% [2]. Thus, it becomes increasingly more important to understand how esophageal cancer develops over time, underscoring the need for identifying molecular mechanisms that drive the development of esophageal cancer.

Esophageal cancer cells preferentially metabolize glucose, and express high levels of glycolytic enzymes such as PKM2 and Lactate dehydrogenase A (LDHA), which contribute to the invasion, metastasis, and poor outcome [3, 4]. In addition, expression of Hexokinase-1 (HK1), another glycolytic enzyme, is associated with disease progression, invasion, and poor survival of patients with ESCC [5]. These studies suggest that cancer metabolism is critical to the development and prognosis of ESCC.

A high rate of glycolysis contributes to the development of ESCC and generates high lactic acid levels in tumor areas [5]. Regulation of the resulting intracellular and extracellular pH changes, which affect the development of cancer, are largely mediated by Vacuolar H^+^-ATPase [6]. Moreover, activity of V-ATPase is sensitive to glucose availability and phosphoinositide 3-kinase (PI3K) signaling [7].

V-ATPase is required for amino acid signaling by interacting with mammalian target of rapamycin complex 1 (mTORC1) [8]. V-ATPase, as a transcriptional effector of E2F1, induces nucleotide loading of Rag GTPases, which promotes the translocation of mTORC1 to the lysosomal surface [8, 9], suggesting a potential role of V-ATPase in nutrient sensitive metabolism. We have previously shown that expression of phosphorylated mTOR and ribosomal S6 protein, which are key anabolic signaling components in response to nutrient levels and cancer metabolism, is associated with poor survival rates of ESCC patients [10, 11]. Because V-ATPase is an mTORC 1 interacting protein, we sought to determine the role of V-ATPase in cancer metabolism in ESCC.

V-ATPase is a ubiquitously expressed ATP-dependent proton pump, and plays a role in maintaining a relatively neutral intracellular pH, an acidic luminal pH, and an acidic extracellular pH [12]. V-ATPase-dependent acidification is necessary for intracellular processes such as protein sorting and trafficking [13]. V-ATPase is present on the plasma membrane of specialized cell types, such as renal proximal tubule epithelial cells, and it is involved in various biological functions including urinary acidification, bone resorption, and tumor cell invasiveness [13], suggesting that V-ATPase has multiple roles in acid secretion, osteoclast function, and tumor metastasis. Structurally, V-ATPase is a large multi-subunit complex consisting of two domains (V0 and V1). The V1 domain includes 8 subunits (A, B, C, D, E, F, G, H), and is responsible for ATP hydrolysis; the V0 domain includes 5 subunits (a, c, c', c'', e), and is involved in proton translocation [14]. The A, C, and G subunits have been implicated in the development of glioma, gastric, and breast cancer and high expression of the A, C, and G subunits is associated with shorter overall survival. [15–17]. The E-subunit forms a heterodimer with the G-subunit. Three EG heterodimers comprise three peripheral stators exposed on the outside of V-ATPase, which regulate assembly and disassembly of the of V-ATPase [18]. Regulated assembly of V-ATPase is a an important mechanism of modulating V-ATPase activity critical to survival and invasion of breast cancer cells [19]. Expression of the V-ATPase V1E1 subunit is markedly increased in breast cancer tissues that also express Rab27B, a member of the RAS oncogene family [20], suggesting a contribution of V-ATPase V1E1 to breast cancer.

Here, we examined the clinical relevance of V-ATPase V1E1, and determined the prognostic impact of increased V-ATPase V1E1 levels on the survival of ESCC patients. In addition, we assessed the effects of V-ATPase V1E1 depletion on esophageal cancer cell viability, aerobic glycolysis, glycolytic gene expression, and oxygen consumption. We analyzed genetic alterations in V-ATPase V1E1 in ESCC using TCGA data and GSEA analysis, and assessed the effects of V-ATPase V1E1 expression on the survival of patients with ESCC.

## RESULTS

### Enhanced expression of V-ATPase V1E1 in ESCC correlates with tumor invasion and lymph node metastasis

**Figure 1:**
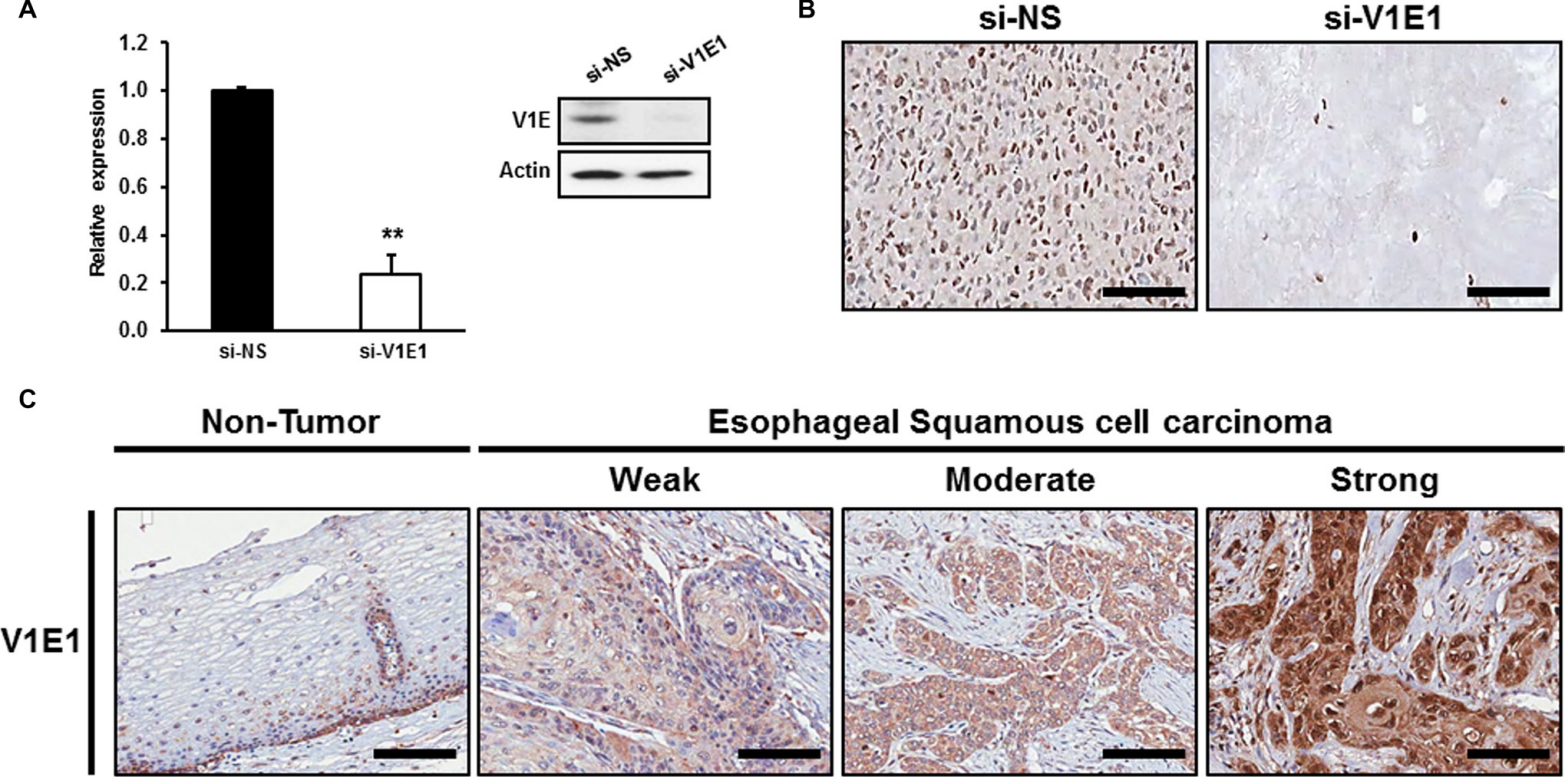
Immunohistochemical analysis of V-ATPase V1E1 in non-tumor esophageal and esophageal squamous cell carcinoma tissues (**A**) Expression levels of V-ATPase V1E1 were determined in TE8 cells using qRT-PCR or western blotting. (**B**) Expression levels of V-ATPase V1E1 were determined in *paraffin*-embedded TE8 *cell block* sections. TE8 cells were transfected with non-silencing siRNA (NS), or si-V1E1 (50 nM) during 72 hr. (**C**) Representative V-ATPase V1E1 immunostaining in non-tumor esophageal or esophageal squamous cell carcinoma tissues with weak, moderate, or strong expression. Original magnification ×200, Scale bars, 100 μm. Values are mean ± SEM. (Student's *t*-test, **P < 0.01).

We determined the expression patterns of V-ATPase V1E1 in esophageal cancer using immunohistochemistry. We first validated the specificity of antibodies by immunostaining sections containing TE8 esophageal cancer cells, in which V-ATPase V1E1 was knocked down or not with siRNA or non-silencing siRNA (control). The antibody used specifically stained V-ATPase V1E1-positive TE8 cells treated with non-silencing siRNA but did not stain V-ATPase V1E1 knockdown cells, which demonstrated the specificity of the antibody (Figure 1A, 1B). We then performed immunohistochemical analysis of esophageal tissues from 160 esophageal cancer patients and 31 normal esophageal tissue samples (Table 1). We found that V-ATPase V1E1 expression correlated with direct tumor invasion (pT stage) (*P* = 0.041), and high expression was significantly more frequent in cases in which lymph node metastasis had occurred (*P* = 0.041) (Table 1). Abundant expression of V-ATPase V1E1 was observed in the cytoplasm of cancer cells, exhibiting more than moderate staining in 48% of samples (77/160) (Table 2). V1E1 was much less frequently expressed in non-tumor esophageal tissues (*P* = 0.017) (Figure 1C and Table 2).

**Table 1:** Relation between the expression of V-ATPase V1E1 and clinicopathologic variables

	Case no. (*n* = 160)	V-ATPase V1E1	*P* value
**Age**			
≥ 65 years	123	63/123 (51.2%)	0.190
< 65 years	37	14/37 (37.8%)	
**Sex**			
Male	155	75/155 (48.4%)	1.000
Female	5	2/5 (40.0%)	
**Tumor size**			
≥ 4.0 cm	90	44/90 (48.9%)	0.874
< 4.0 cm	70	33/70 (47.1%)	
**Differentiation**			
W/D	25	10/25 (40.0%)	
M/D	106	52/106 (49.1%)	0.654
P/D	29	15/29 (51.7%)	
**TNM Stage**			
I	27	11/27 (40.7%)	
II	53	22/53 (41.5%)	0.348
III	60	34/60 (56.7%)	
IV	20	10/20 (50.0%)	
**Tumor invasion**			
T1	34	12/34 (35.3%)	
T2	28	12/28 (42.9%)	0.041*
T3	90	48/90 (53.3%)	
T4	8	5/8 (62.5%)	
**LN metastasis**			
Negative	62	24/62 (38.7%)	0.041*
Positive	98	53/98 (54.1%)	
**Distant metastasis**			
Absent	138	65/138 (47.1%)	0.525
Positive	22	11/22 (52.4%)	

* *P* < 0.05.

**Table 2:** Results of the immunohistochemical analysis of V-ATPase V1E1 expression in normal and ESCC tissues

	Normal esophagus	Chronic esophagitis	ESCC	*P* value
**High expression of V-ATPase V1E1**	3/20 (15.0%)	4/11 (36.4%)	77/160 (48.1%)	0.017*

* *P* < 0.05.

### High expression of V-ATPase V1E1 is associated with poor prognosis especially in early stage of ESCC

**Figure 2:**
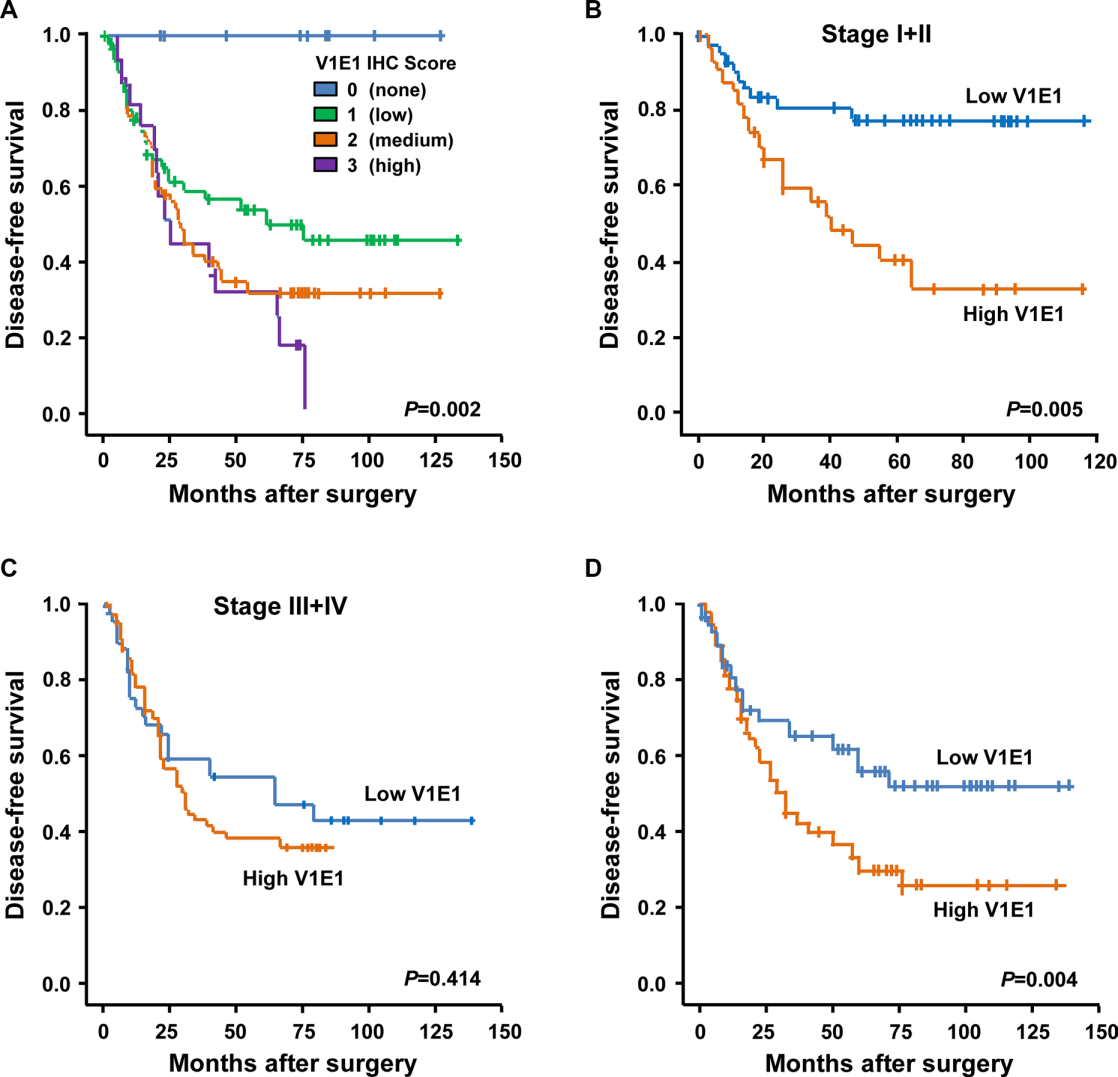
Kaplan-Meier survival curves for disease-free survival according to the results of V-ATPase V1E1 immunostaining (**A**) Kaplan-Meier curves showing disease-free survival among patients with ESCC on the basis of V-ATPase V1E1 expression status; V-ATPase V1E1 (IHC score = 0, *n* = 12; IHC score=1, *n* = 72; IHC score =2, *n* = 60; IHC score =3, *n* = 16). (**B**) Disease-free survival among patients with ESCC on the basis of V-ATPase V1E1 expression status at stage I + II (low V1E1, IHC score < 2, *n* = 47; high V1E1, IHC score ≥ 2, *n* = 34). (**C**) Disease-free survival among patients with ESCC on the basis of V-ATPase V1E1 expression status at stage III + IV (low V1E1, IHC score < 2, *n* = 37; high V1E1, IHC score ≥ 2, *n* = 42). (**D**) Disease-free survival among patients with ESCC on the basis of V-ATPase V1E1 expression status; low V1E1 (IHC score < 2, *n* = 84) and high V1E1 (IHC score ≥ 2, *n* = 76). *P* values are from Log-Rank test.

We assessed possible associations between V-ATPase V1E1 expression and patient survival. Kaplan-Meier survival analysis showed a dramatic correlation between V-ATPase V1E1 levels and patient survival (Figure 2A). Patients with higher IHC scores of V-ATPase V1E1 had reduced disease-free survival (*P* = 0.002) and shorter overall survival (*P* = 0.017) (Figure 2A and Supplementary Figure S1A). In particular, all patients showing no V-ATPase V1E1 expression survived without recurrence (Figure 2A). We assessed survival relative to tumor grade and V-ATPase V1E1 expression. For this analysis patients were grouped into early stage (stage I + II) and late stage (stage III + IV) disease. High V-ATPase V1E1 levels were more significantly associated with reduced disease-free survival in early-stage ESCC patients (*P* = 0.005) than in late-stage patients (*P* = 0.414) (Figure 2B, 2C). These results suggest that expression of V-ATPase V1E1 in early stage disease is more relevant to adverse clinical outcomes than expression in advanced stage disease. This conclusion is supported by the fact that high expression of V-ATPase V1E1 was significantly associated with reduced disease-free survival (*P* = 0.004; Figure 2D) and reduced overall survival (Supplementary Figure S1B).

### V-ATPase V1E1 is an independent prognostic factor in ESCC

To determine whether V-ATPase V1E1 was an independent prognostic factor in ESCC, we performed multivariate analysis of V-ATPase V1E1 expression with respect to disease free survival rates of esophageal cancer patients using Cox proportional-hazard regression. Patient age, TNM stage, history of chemotherapy and radiation therapy, and V-ATPase V1E1 expression data were entered into a Cox proportional-hazard model. We found that V-ATPase V1E1 protein expression was an independent prognostic factor for disease-free survival (HR, 1.748; 95% CI, 1.1–2.8; *P* = 0.018) (Table 3). TNM stage III (HR, 4.325; 95% CI, 1.7–11.1; *P* < 0.003), (HR, 7.017; 95% Cl, 2.1–22.9; *P* = 0.002) and stage IV (HR, 7.498; 95% CI, 2.7–20.7; *P* < 0.001), (HR, 9.556; 95% CI, 2.7–34.0; *P* = 0.001) were also independent prognostic factors for disease-free and overall survival (Table 3). Moreover, disease-free (HR, 0.722; 95% CI; 0.6–0.9, *P* = 0.004) and overall survival (HR, 0.732; 95% CI; 0.6–0.9, *P* = 0.010) of patients with recurrent esophageal cancer treated with radiation therapy was poorer in patients with high V-ATPase V1E1 expression (Table 3). These results showed that high levels of V-ATPase V1E1 expression correlated with TNM stage and correlated with recurrent esophageal cancer treated with radiation therapy.

**Table 3:** Multivariate Cox regression analysis of V-ATPase V1E1 and other covariates for ESCC patients' survival rate

Predictors	Disease-free survival		Overall survival	
	HR (95% CI)	*P* value	HR^1^ (95% CI^2^)	*P* value
**Age**				
0–65	1.00		1.00	
65+	1.103 (0.6–1.9)	0.720	1.025 (0.6–1.9)	0.935
**TNM Stage**				
I	1.00		1.00	
II	2.320 (0.9–6.1)	0.093	3.222 (0.9–11.0)	0.062
III	**4.325 (1.7–11.1)**	< 0.003**	**7.017 (2.1–22.9)**	0.002**
IV	**7.498 (2.7–20.7)**	< 0.001**	**9.556 (2.7–34.0)**	0.001**
**Chemotherapy**				
Absent	1.00		1.00	
Positive	1.062 (0.8–1.4)	0.632	1.116 (0.6–1.5)	0.410
**Radiation therapy**				
Absent	1.00		1.00	
Positive	**0.722 (0.6–0.9)**	0.004**	**0.732 (0.6–0.9)**	0.010*
**V-ATPase V1E1**				
Negative	1.00		1.00	
Positive	**1.748 (1.1–2.8)**	0.018*	1.394 (0.9–2.3)	0.188

1 HR, Hazard Ratio. *P < 0.05, **P < 0.01.

2 CI, Confidence interval. *P < 0.05, **P < 0.01.

### Depletion of V-ATPase V1E1 reduces proliferation in TE8 esophageal cancer cells

**Figure 3:**
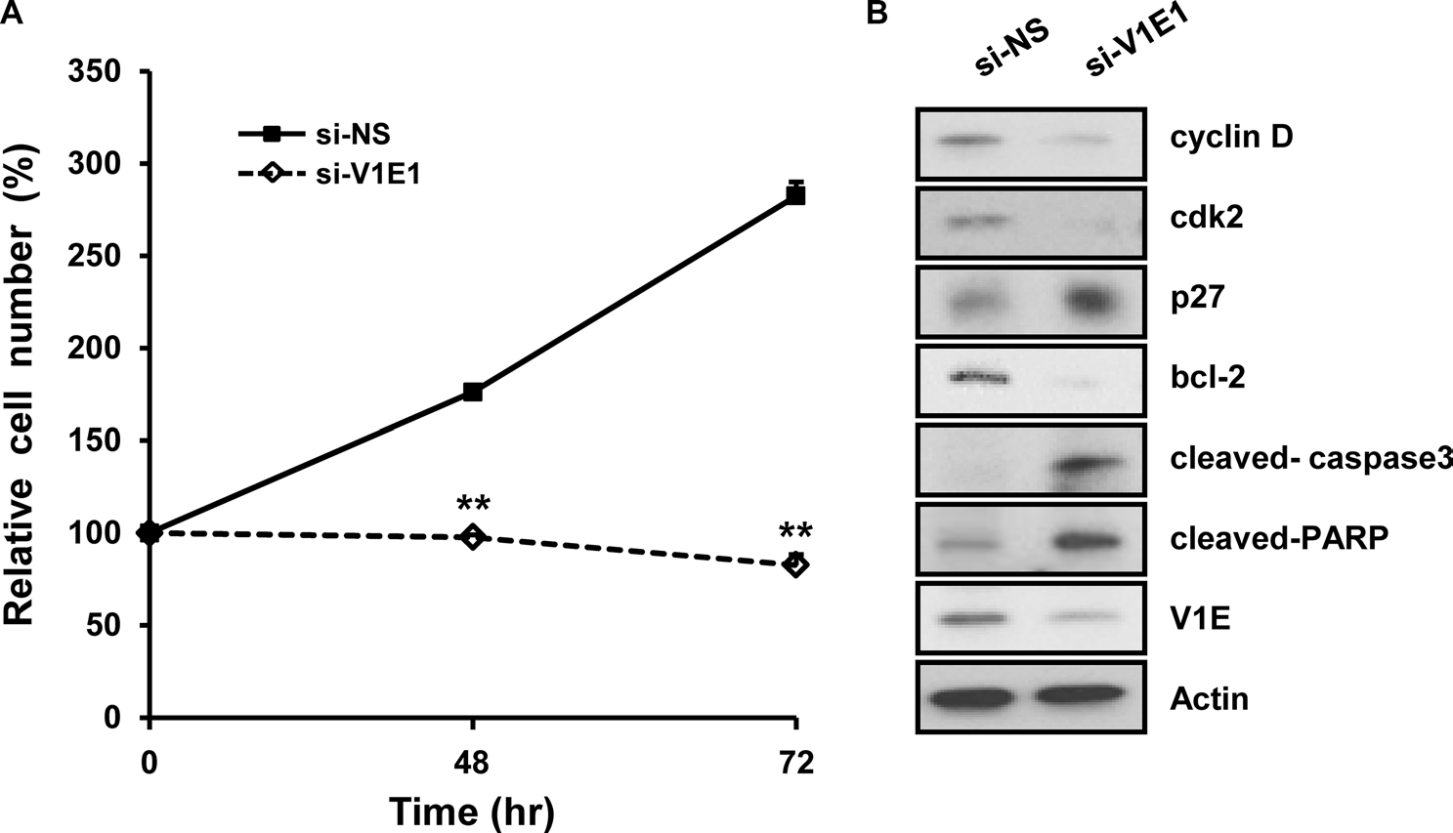
Depletion of V-ATPase V1E1 significantly reduces viability of TE8 esophageal cancer cells TE8 cells were transfected with non-silencing siRNA (NS), or si-V1E1 during the indicated times. (**A**) Cell viability was analyzed by trypan blue assay. TE8 cells were transfected with non-silencing siRNA (NS) or si-V1E1 (50 nM). (**B**) Cell lysates were analyzed by immunoblot using the indicated antibodies. Assays and blots are representative of three independent experiments. Values are means ± SEM. (ANOVA, **P < 0.01).

Based on the fact that the expression of V-ATPase is associated with cell growth capacity, which affects cell size and viability [15, 17], we determined whether depletion of V-ATPase V1E1 affected viability of esophageal cancer cells. TE8 esophageal cancer cells exhibit high colony formation efficiency and overexpress epidermal growth factor receptors (EGFRs) [21]. However, knockdown of V-ATPase V1E1 resulted in decreased proliferation of TE8 esophageal cancer cells (Figure 3A). Moreover, depletion of V-ATPase V1E1 resulted in reduced expression of positive cell cycle regulators, such as cyclin D and cyclin-dependent kinase 2 (cdk2) (Figure 3B), indicating G1 cell cycle arrest. We then determined whether expression of V-ATPase V1E1 affected apoptosis of TE8 esophageal cancer cells. Reducing levels of V-ATPase V1E1 resulted in reduced expression of Bcl-2 and cell survival-related proteins (Figure 3B). Moreover, levels of apoptotic proteins including cleaved caspase 3 and Poly [ADP-ribose] polymerase 1 (PARP) were increased substantially by V-ATPase V1E1 depletion (Figure 3B). Thus, these results suggest that reducing levels of V-ATPase V1E1 decreases esophageal cancer cell survival by inhibiting cell cycle regulators and increasing expression of apoptosis-related proteins.

### Knockdown of V-ATPase V1E1 attenuates cell motility and focal adhesion in TE8 esophageal cancer cells

**Figure 4:**
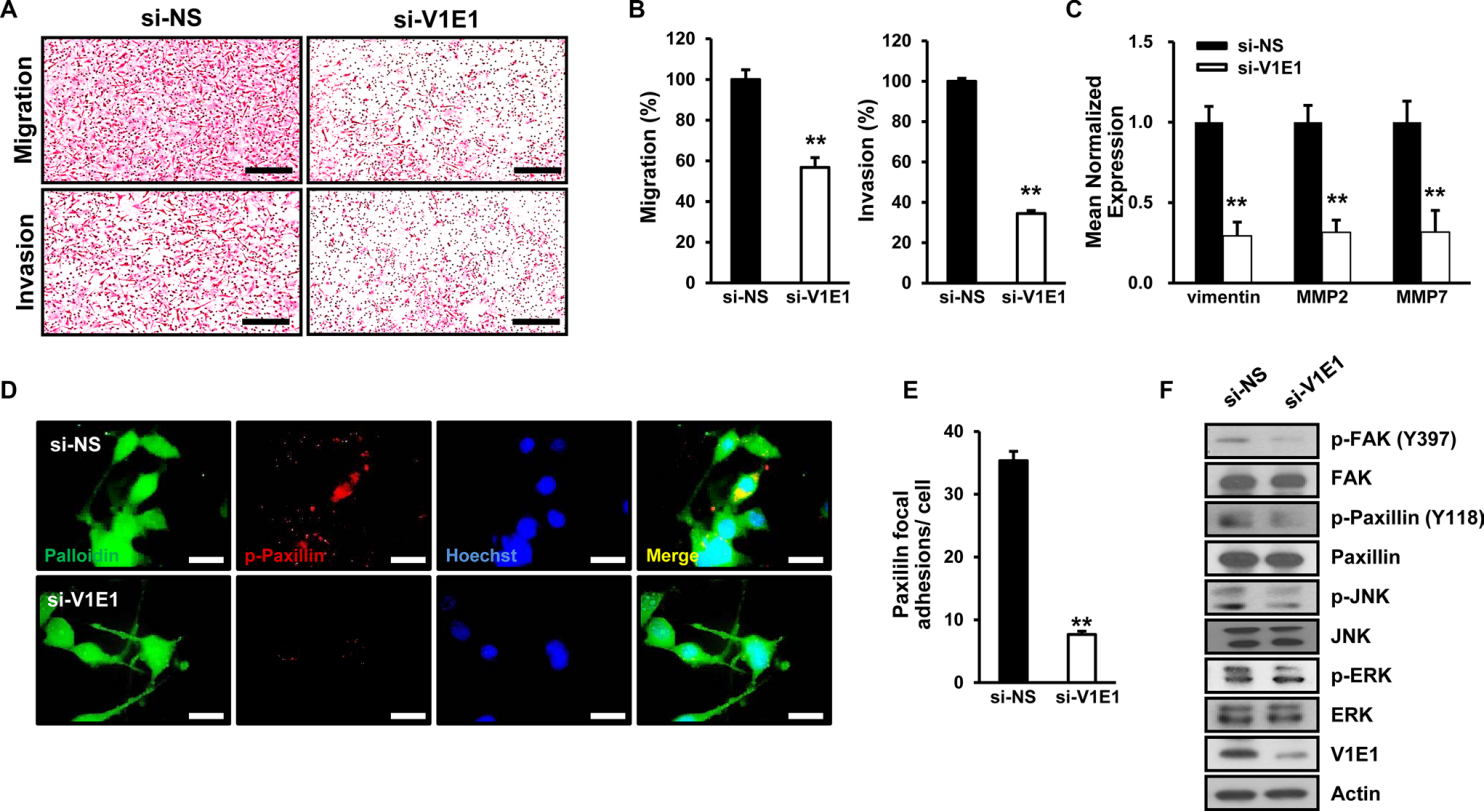
Depletion of V-ATPase V1E1 inhibits migration, invasion, and formation of focal adhesions by TE8 esophageal cancer cells TE8 cells were transfected with non-silencing siRNA (NS) or si-V1E1, and then evaluated by (**A**, **B**). transwell migration assay or invasion assay. Cells were fixed and stained with hematoxylin and eosin and the slides of filters of migrated cells or invaded cells were scanned using an Aperio scanner (original magnification ×100). Scale bars, 400 μm. (**C**) qRT-PCR analysis shows low levels of vimentin, MMP2, and MMP7 expression in V1E1-depleted cells. (**D**) TE8 cells were immunostained with antibody to p-paxillin and rhodamine-labeled secondary antibody, Hoechst, and Alexa 488 phalloidin, and then viewed by fluorescence microscopy. Scale bars, 10 μm. (**E**) The numbers of paxillin focal adhesions per cell were determined. (**F**) Cell lysates were analyzed by immunoblot using the indicated antibodies. Assays and blots are representative of three independent experiments. Values are means ± SEM. (Student's *t*-test, **P < 0.01).

As tumor invasion and lymph node metastasis are important features of ESCC, we examined whether depletion of V-ATPase V1E1 influenced migration and invasion by TE8 esophageal cancer cells. Reducing levels of V-ATPase V1E1 resulted in suppression of migration and invasion by TE8 esophageal cancer cells, indicating the significance of V-ATPase V1E1 to metastatic properties of esophageal cancer cells (Figure 4A, 4B). In addition, V-ATPase V1E1 depletion led to reduced expression of Vimentin, Matrix Metalloproteinases 2 (MMP2), and Matrix Metalloproteinases 7 (MMP7), which mediate epithelial-to-mesenchymal transition (EMT) (Figure 4C). EMT is the first step in the process of metastasis, and converts the adherent phenotype of epithelial cancer cells to an invasive, migratory mesenchymal phenotype [22].

Recent studies have shown that metastatic ESCC are associated with overexpression of focal adhesion kinase (FAK) [23]. Based on this finding, we examined whether V-ATPase V1E1 knockdown affected focal adhesion of TE8 esophageal cancer cells. V-ATPase V1E1 depletion led to pronounced inhibition of focal adhesion formation as revealed by less densely stained phosphorylated paxillin, which mediates the interaction between the actin cytoskeleton and integrin (Figure 4D, 4E). To further determine the signaling components underlying V-ATPase V1E1-mediated focal adhesion and invasion, we assessed the status of signaling pathways that mediate cell motility. Knockdown of V-ATPase V1E1 resulted in inhibition of phosphorylation of extracellular signal regulated kinase (ERK) and c-Jun N-terminal kinase (JNK), which are downstream regulators of FAK in cellular motility (Figure 4F). These data suggest that V-ATPase V1E1 positively regulates cell motility and formation of focal adhesions through FAK and its known downstream effectors, such as ERK and JNK [24].

### Reducing levels of V-ATPase V1E1 attenuates glycolysis in TE8 esophageal cancer cells

**Figure 5:**
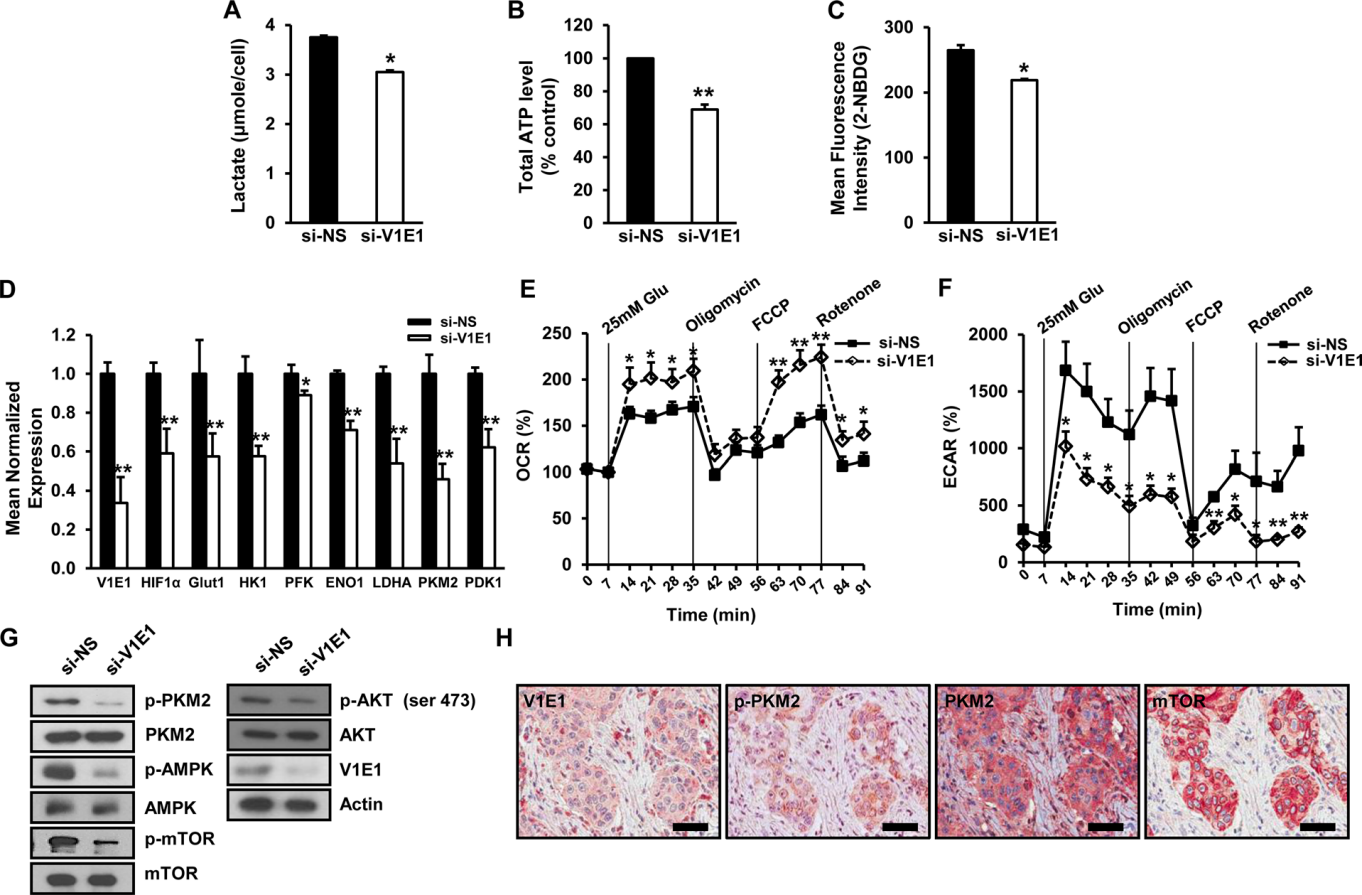
Depletion of V-ATPase V1E1 attenuates glycolysis in TE8 esophageal cancer cells The effects of V-ATPase V1E1 knockdown on the levels of (**A**). Lactate, (**B**) ATP, and (**C**). glucose uptake. TE8 cells were transfected with non-silencing siRNA (NS), or si-V1E1 for 72 hr and then treated with 10 μM 2-NBDG as a fluorescent indicator for direct glucose uptake. Glucose uptake was analyzed by flow cytometry. (**D**) Gene expression of selected glycolytic enzymes and glucose transport proteins in V-ATPase V1E1-depleted cells analyzed by qRT-PCR. (**E**) Oxygen consumption (OCR) or (**F**). extracellular acidification rate (ECAR) in V-ATPase V1E1 depleted cells compared to cells treated with NS (non-silencing siRNA) in response to indicated drugs. Cells were starved of glucose for 1 hr prior to initiation of the experiment. Drugs including 25 mM glucose, 1 mM oligomycin, 300 nM FCCP, and 100 nM rotenone were added at indicated time points. Experiments were conducted in the absence of sodium pyruvate. (**G**) Expression of indicated proteins in TE8 cell lysates treated with non-silencing siRNA (NS) or si-V1E1. (**H**) The concomitant expression of V1E1, p-PKM2, PKM2, and mTOR in ESCC tissues. Original magnification ×200, Scale bars, 100 μm. Assays and blots are representative of three independent experiments. Values are means ± SEM (ANOVA, *P < 0.05, **P < 0.01).

Recent studies have shown that tumorigenesis and metastasis of ESCC are dependent on glycolysis and on increased levels of the glycolytic byproduct, lactate [4]. We measured levels of lactate and ATP, major sources of energy generated from aerobic glycolysis, in TE8 cells after V-ATPase V1E1 knockdown. The levels of lactate and ATP were markedly decreased in V-ATPase V1E1 depleted cells compared to cells treated with non-silencing siRNA (Figure 5A, 5B), indicating that V-ATPase V1E1 acts to shift metabolic flux toward aerobic glycolysis and away from mitochondrial respiration. Furthermore, V-ATPase V1E1 depletion led to suppression of glucose uptake (Figure 5C), which is linked to reduced levels of lactate in V-ATPase V1E1 knockdown cells. Given that glucose uptake is mainly mediated by glucose transporter 1 (Glut1) in cancer cells, and that Glut1 expression is controlled by hypoxia inducible factor 1a (HIF-1α) [25], we examined expression of Glut1 and HIF-1α target genes in V-ATPase V1E1 depleted cells. V-ATPase V1E1 depletion resulted in decreased expression of glycolytic enzymes, Glut1, PKM2, and LDHA (Figure 5D), which are key drivers of the metabolic switch from oxidative phosphorylation to aerobic glycolysis. To further investigate whether V-ATPase V1E1 regulates this metabolic shift through modulating oxygen consumption rate (OCR) and extracellular acidification rate (ECAR), we treated cells after V-ATPase V1E1 knockdown with 25 mM glucose in the absence or presence of carbonyl cyanide p-trifluoromethoxyphenylhydrazone (FCCP), which stimulates respiration in mitochondria, rotenone, which blocks respiration by interfering with the electron transport chain in mitochondria, or oligomycin, which also blocks respiration by interfering with the electron transport chain [26]. Mitochondrial oxidative phosphorylation (OXPHOS) was enhanced in V1E1 depleted cells after treatment with FCCP, and OCR was enhanced in V-ATPase V1E1 depleted cells after treatment with rotenone (Figure 5E). These results suggest that reducing levels of V-ATPase V1E1 increased the rate of mitochondrial respiration through glucose oxidation. Conversely, ECAR was reduced in V-ATPase V1E1 depleted cells after treatment with oligomycin, rotenone, or FCCP (Figure 5F). These data suggest that depletion of V-ATPase V1E1 led to increased glucose oxidation in TE8 esophageal cancer cells by inhibiting glycolysis (Figure 5E, 5F).

Glycolysis of cancer cells is mediated by the PI3K/AKT pathway [27]. Activated AKT induces glucose uptake and phosphorylation of PKM2, leading to enhanced expression of glycolytic enzymes [28]. Considering that phosphorylation of PKM2 at Tyr 105 disrupts the formation of active tetrameric PKM2 by releasing the cofactor fructose-1,6-bisphosphate (FBP) [29] and favoring its nuclear translocation to induce glycolytic enzyme expression [30], we examined the effects of V-ATPase V1E1 depletion on phosphorylation of AKT and PKM2. V-ATPase V1E1 depletion resulted in decreased phosphorylation levels of AKT at ser473 and of PKM2 at Tyr105, while total AKT and PKM2 levels remained unchanged (Figure 5G), suggesting that V1E1 promotes glycolysis through AKT and PKM2. To determine whether the expression of V-ATPase V1E1 was associated with PKM2, we measured the concomitant expression of V-ATPase V1E1, p-PKM2, PKM2, and mTOR in esophageal cancer tissues (Figure 5H). Esophageal cancer tissues were serially stained with each antibody. Between each round of staining, each slide was image-captured to generate a virtual microscope file, and then antibodies were stripped from the slide, and staining with the next antibody was performed. With this novel multiplex immunohistochemistry, we found that there was significant overlap among expression of these proteins in esophageal cancer tissues. Together, these results indicate that V-ATPase V1E1 is critical for aerobic glycolysis, and that its presence is required for regulation of the levels of ATP, lactate synthesis, expression of glycolytic enzymes, and signaling related to glucose metabolism in TE8 esophageal cancer cells.

### Genetic alterations in V-ATPase V1E1 are frequent in ESCC, and V-ATPase V1E1 expression is associated with a gene-expression signature of cancer metabolism

**Figure 6:**
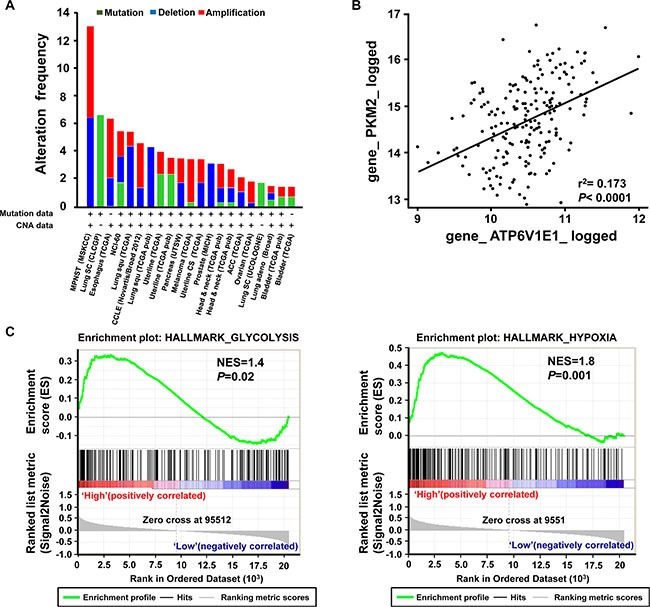
ESCC tissues exhibit enhanced genomic alterations of V-ATPase V1E1 and gene signatures related to cancer metabolism (**A**) Frequency of copy number alterations and mutations of V-ATPase V1E1 across several tumor types. (**B**) Correlation between V1E1 and PKM2 mRNA expression levels in ESCC (*n* = 1176). (A, B) The results shown here are based upon data generated by the TCGA Research Network (Available online: http://cancergenome.nih.gov/). (**C**) GSEA analysis on expression data set from V1E1-positive (*n* = 11) and V1E1-negative (*n* = 8). ESCC tissues showing enriched gene sets related to cancer metabolism. Normalized Enrichment Scores (NES) and *P* values are presented.

The results presented above suggested that V-ATPase V1E1 plays a critical role in cancer metabolism in esophageal cancer cells. To determine whether these results are applicable to ESCC patients, we investigated the functional phenotypes associated with elevated V-ATPase V1E1 expression in ESCC patients using bioinformatics analyses. First, we retrieved whole exome sequencing and mRNA expression data of 1176 ESCC patients from the Cancer Genome Atlas (TCGA) database. Genetic alterations, especially amplification of V-ATPase V1E1, were frequently found in ESCC compared with other types of cancer. Only Malignant Peripheral Nerve Sheath Tumors (MPNST) and lung cancers exhibited more frequent alterations, underscoring the clinical significance of V-ATPase V1E1 in ESCC (Figure 6A). In addition, a strong positive correlation between V-ATPase V1E1 and PKM2 mRNA levels was observed in the ESCC patient cohort (*n* = 196) (*R*^2^ = 0.173, Figure 6B). This finding is consistent with our result showing that V-ATPase V1E1 enhanced glycolysis through increasing expression and phosphorylation of PKM2 in ESCC. To assess functional implications of V-ATPase V1E1 in ESCC patients, we selected the V-ATPase V1E1-enriched population from TCGA database and performed Gene Set Enrichment Analysis (GSEA) on this population. We found that gene expression signatures related to cancer metabolism, including hypoxia (NES = 1.8) and glycolysis signaling (NES = 1.4), were enriched in patients expressing high V1E1 levels (Figure 6C). These results provide further evidence that V1E1 is involved in facilitating tumor progression by upregulating expression of glycolysis-related genes.

### High expression of V-ATPase V1E1 and phosphorylation of PKM2 predicts poorer prognosis in ESCC

**Figure 7:**
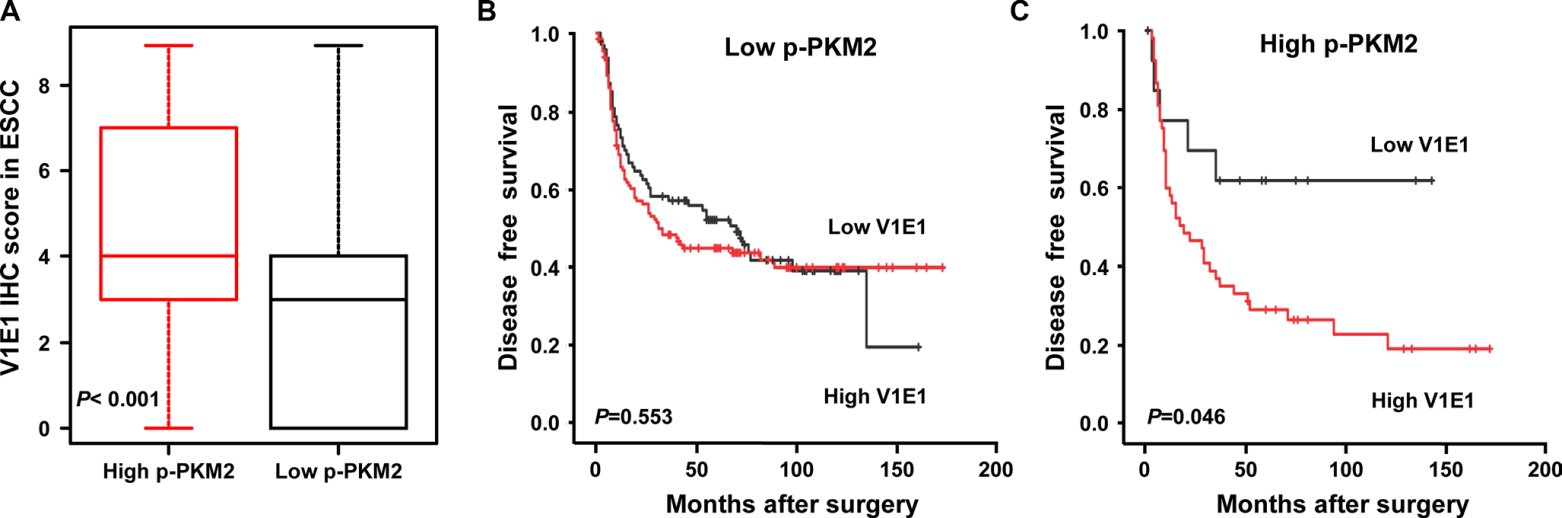
Kaplan-Meier curves showing disease free survival according to the expression of V-ATPase V1E1 and p-PKM2 (**A**) Box plot distribution of V1E1 immunohistochemical scores in ESCC patients with high p-PKM2 expression (IHC score ≥ 3, *n* = 66) or low p-PKM2 expression (IHC score ≤ 2, *n* = 236). Spearman's rank correlation test. Plots were scaled by dividing each median value. (**B**) Disease-free survival curve among patients with ESCC expressing low p-PKM2 on the basis of V-ATPase V1E1 expression status (low V1E1, IHC score ≤ 2, *n* = 99; high V1E1, IHC score ≥ 3, *n* = 137). (**C**) Disease-free survival curve among patients with ESCC expressing high p-PKM2 on the basis of V-ATPase V1E1 expression status (low V1E1, IHC score ≤ 2, *n* = 14; high V1E1, IHC score ≥ 3, *n* = 52). Log-Rank test.

As shown above, V-ATPase V1E1 regulates expression and phosphorylation of PKM2, a key player in the “Warburg effect”, in esophageal cancer cells. To assess the clinical relevance of this finding, we performed an immunohistochemical examination of the protein levels of V-ATPase V1E1 and p-PKM2 in cancer tissues from a distinct ESCC cohort (*n* = 302) (Supplementary Table S1). Our results substantiated the observation that V-ATPase V1E1 was an adverse prognostic indicator. To assess a possible correlation between V-ATPase V1E1 and p-PKM2, we performed normality testing to determine whether our V-ATPase V1E1 expression data set exhibited a normal distribution using a Shapiro-Wilk test, and applied our data to a Spearman rank correlation test. This analysis showed a strong correlation between levels of V-ATPase V1E1 and p-PKM2 (*P* < 0.001) (Figure 7A). The prognostic value of V-ATPase V1E1 expression in ESCC was lost in patients whose cancer cells expressed little or no p-PKM2 (Figure 7B, 7C), indicating that the impact of V-ATPase V1E1 on patient survival was dependent on p-PKM2 expression. Thus, this immunohistochemical analysis of ESCC tissues provides evidence that V-ATPase V1E1 facilitates progression of ESCC by regulating cancer metabolism, especially glycolysis.

## DISCUSSION

V-ATPase assembly is regulated by glucose [7], and is required for the activation of Rag GTPases and mTOR, which are critical for amino acid sensing. In addition, V-ATPase expression is known to affect lysosomal recruitment of E2F1-induced mTORC1 in response to nutrient availability [8, 9]. Consistent with the role of V-ATPase on nutrient sensing with mTORC1, malfunction or overexpression of V-ATPase has been reported in breast cancer and other diseases, including inherited distal renal tubular acidosis, sensorineural deafness, and osteopetrosis [13]. In this study, we have found that V-ATPase V1E1 expression is associated with shortened survival of patients with ESCC. We further found that V-ATPase V1E1 protein expression increased in advanced ESCC in concert with increasing T and N stage parameters. Consistent with our findings, V-ATPase, as a multi-subunit enzyme, has been reported to be overexpressed in other highly invasive tumors, such as ovarian cancer and glioma, and to correlate with metastasis and poor prognosis [16, 31].

Our results demonstrated that depletion of V-ATPase V1E1 induced cell cycle arrest by reducing the expression of cyclin D1 and cdk2, and increasing expression of apoptosis-related proteins. Relevant to this, a recent study reported that lowering levels of V-ATPase subunit V0d1 decreases cell-cycle progression of breast cancer cells that express GFP-Rab27B [20]. In addition, knockdown of V-ATPase subunit V1G or treatment with the V-ATPase inhibitor, archazolid, results in apoptosis in glioblastoma and bladder cancer [16, 32]. These studies, in addition to ours, suggest that V-ATPase affects cell cycle and apoptosis in cancer cells. It will be important to determine how V-ATPase V1E1 controls cell cycle related proteins and regulates the expression of apoptotic proteins in esophageal cancer cells.

The highly invasive and metastatic behavior of esophageal cancer contributes to poor survival of patients with ESCC [1]. In line with this, we showed that knockdownof V-ATPase V1E1 resulted in a reduction of migration and invasion of esophageal squamous cell carcinoma cells. In addition, recent studies showed that the expression of V-ATPase subunits V1A, V1G and V0a are associated with metastasis of ovarian cancer, glioma, and gastric cancer [15, 16, 31]. Inhibition of V-ATPase expression using siRNA or V-ATPase inhibitors suppresses cell growth and metastasis in hepatocellular carcinoma and breast cancer [32, 33]. We further found that depletion of V-ATPase V1E1 led to reduced expression of vimentin, MMP2, and MMP7, which are critical for metastasis of esophageal cancer cells. Thus, reducing the levels of V-ATPase would likely suppress migration of esophageal cancer cells by suppressingthe expression of Vimentin and MMP family proteins, which would be applicable for the development of potential therapeutic strategies against ESCC.

We found that knockdown of V-ATPase V1E1 led to a reduction in phosphorylation of FAK and paxillin, which enhanced focal adhesion assembly. Consistent with this, Schempp et al demonstrated that Archazolid, which binds subunit c in the V0 domain of V-ATPase and thereby inhibits its activity, impairs FAK activity in human urinary carcinoma cells and decreases metastasis *in vivo*, suggesting the functional relevance of V-ATPase subunit V0C in focal adhesion and migration [32]. In addition, esomeprazole, a H^+^/K^+^ ATPase inhibitor, has been shown to reduce tumor cell survival, metastatic potential, and sensitivity to chemotherapy in esophageal cancer cell lines [34]. Thus, these findings and ours indicate that V-ATPase is critical for metastasis of ESCC.

Overexpression of Glut1 and HK-II using 18F-fluorodeoxyglucose positron emission tomography (FDG-PET) has been shown to correlate with hexokinase activity and glucose uptake in esophageal cancer [35]. In our data, lowering the levels of V-ATPase V1E1 resulted in decreased lactate levels and ATP production in TE8 cells. It would be of interest to determine how V-ATPase V1E1 regulates aerobic glycolysis in the context of tumor invasion and migration in ESCC. Intriguingly, the V-ATPase V1E1 subunit binds directly to aldolase, a key glycolytic enzyme, which converts fructose 1,6-bisphosphate into the triose phosphates dihydroxyacetone phosphate (DHAP) and glyceraldehyde 3-phosphate [18]. Thus, it is possible that V-ATPase V1E1 affects glycolysis through its association with aldolase.

Our results also showed that lowering the levels of V-ATPase V1E1 resulted in suppressed mRNA levels of glycolytic enzymes such as HIF-1α and its target genes, such as Glut1 and LDHA, as well as decreased glucose uptake in TE8 cells. These findings suggest that V-ATPase V1E1 affects glycolysis by regulating expression of glycolytic enzymes and glucose uptake, a process for providing energy substrates to esophageal cancer cells. Given that LDHA expression is associated with the progression of ESCC [4], and its reduction induces enhancement of oxygen consumption [36], V-ATPase V1E1-mediated glycolysis may be critical for the development of metastatic ESCC.

We found that V-ATPase V1E1 depletion induced a metabolic switch that led to decreased phosphorylation of PKM2 Tyr 105. PKM2 is a key molecule that induces glucose uptake and expression of glycolytic enzymes [30]. Moreover, the Cancer Genome Atlas (TCGA) data and Gene Set Enrichment Analysis (GSEA) indicated that genomic alteration of V-ATPase V1E1 was frequently observed in ESCC, and V-ATPase V1E1 expression was significantly associated with gene signatures of cancer metabolism. Consistent with this, high expression of both V-ATPase V1E1 and p-PKM2 was associated with poorer prognosis in ESCC, implicating V-ATPase V1E1 in deregulation of glucose metabolism in the development of ESCC. Similar to our results, high expression of PKM2 and vascular endothelial growth factor-C, an important molecule in angiogenesis and lymphangiogenesis, correlated significantly with poor prognosis in breast cancer [37]. Therefore, we might expect that alterations in expression of both V1E1 and p-PKM2 would exert compound effects on the prognosis of ESCC. It will be of interest to determine how V-ATPase V1E1 regulates phosphorylation of PKM2 and contributes to altered expression of glucose metabolism-related genes in esophageal cancer.

Our current data demonstrate that the expression of V-ATPase V1E1 is associated with poor clinical outcome of patients with early stage of ESCC. Our findings showed that V-ATPase V1E1 affected migration, invasion, and aerobic glycolysis of esophageal cancer cells and increased phosphorylation of PKM2. Our multivariate analysis and bioinformatics analysis indicated that V-ATPase V1E1 expression was a significant risk factor for survival in ESCC, and had significant prognostic value. Our findings suggest that therapeutic strategies designed to modulate V-ATPase V1E1 expression may be beneficial for the treatment of esophageal squamous cell carcinoma.

## MATERIALS AND METHODS

### Patients and tissue samples

Informed consent for the use of surgical specimens for research purposes was obtained from all patients. The surgical specimens consisted of 160 cases of esophageal squamous cell carcinoma and 31 cases of non-tumor esophageal tissues. The data were procured from surgical pathology files kept at the Department of Pathology in Samsung Medical Center, Seoul, Korea, from 1996 to 2007. Pathologic features of specimens were classified based on the seventh edition of the tumor-nodes-metastasis (TNM) classification (Union for International Cancer Control (UICC)). All archival materials were routinely fixed in 10% neutral buffered formalin and embedded in paraffin. The clinicopathological features of the 160 esophageal squamous cell carcinomas are shown in Table 1. A validation set of 302 cases of ESCC tissues was prepared using samples from the same institution (Supplementary Table S1).

### Immunohistochemical staining procedure

Immunostaining was performed using antibodies against V-ATPase subunit V1E1 (Cat. HPA029196) from Sigma, USA. Tissue sections were deparaffinized with xylene and rehydrated; antigen retrieval was performed by microwaving these sections in Tris-EDTA buffer (10 mM Tris, 1 mM EDTA, pH 9.5) for 20 min. Slides were blocked with peroxidase-blocking solution (Dako REAL™Peroxidase-Blocking Solution; Dako, CA, USA) for 8 minutes, incubated with primary antibodies (1:100 dilution), and rinsed with washing buffer (0.1% Tween 20 in distilled water). Sections were further incubated with DAKO REAL EnVision/HRP, Rabbit/Mouse (Envision) detection reagent. After rinsing, the chromogen was developed. Slides were then counterstained with Meyer's hematoxylin, dehydrated, and mounted with Canada balsam for examination.

### Evaluation of results of immunohistochemical staining

We used the scoring method of Sinicrope et al. [38] to evaluate both the intensity of staining and the proportion of cells stained with V-ATPase subunit V1E1. The staining intensity was scored as: 1 (weak), 2 (moderate), or 3 (strong). The proportion of positive cells was classified into one of the five categories: 1 (< 5%), 2 (5%–25%), 3 (26%–50%), 4 (51%–75%), or 5 (> 75%). Scores for both parameters (staining intensity and proportion of positive cells) were multiplied to generate an aggregate immunohistochemistry score for V-ATPase V1E1 in each sample. For example, if the reactivity score was 3 (strong) and the proportion of positive cells was 3 (26%–50% × 3 = 9. Each lesion was separately examined and scored by two pathologists (SHK and CKP). The pathologists discussed any cases showing a discrepancy in scores until a consensus was reached.

### Cell culture and transfection

The human esophageal squamous carcinoma TE8 cell line was obtained from RIKEN (Saitama, Japan). Cells were cultured in RPMI-1640 (Welgene, Korea) containing 10% FBS (Invitrogen, CA, USA). To suppress V-ATPase V1E1 expression, cells were transfected with each siRNA using G-fectin (GP-2000). The sequence of the siRNA oligonucleotide was as follows: si-ATP6V1E1 5′-CAGATGTCCAATTTGATGAAT-3′. A non-silencing control siRNA (Qiagen, Germany) was also used. Cells were harvested 72 h after transfection.

### RNA purification and quantitative RT-PCR

RNA Purification and Quantitative RT-PCR were conducted as previously described [39]. Primers used for the quantitative RT-PCR are described in Supplementary Table S2.

### Western blotting

Western blotting was conducted as previously described [40] using antibodies from the following sources: V-ATPase V1E1 (Cat. HPA029196) from Sigma, USA; p-JNK (Thr183/Tyr185, Cat. 9251), extracellular signal-regulated kinase (ERK) (Cat. 9102), p-ERK (Cat. 9101), p-paxillin (Tyr 118, Cat. 2541), cleaved-caspase3 (Cat. 9664), cleaved-PARP (Cat. 5625), AKT (Cat. 9272), p-AKT (Cat. 9271), PKM2 (Cat. 4053), p-PKM2 (Cat. 3827), AMPK (Cat 2532), p-AMPK (Cat 2535), mTOR (Cat 2972), and p-mTOR (Cat 2974) from Cell Signaling Technology; c-Jun N-terminal kinase (JNK) (Cat. sc6254), cyclin D (Cat. sc397), cdk2 (Cat. sc163), p27 (Cat. sc528), p-FAK (Tyr 397, Cat. sc1688), focal adhesion kinase (FAK) (Cat. sc558), Bcl-2 (Cat. Sc7381), and β-actin (Cat. sc1616) from Santa Cruz Biotechnology, CA, USA; paxillin (BD Biosciences, Cat. BD610619, NJ, USA).

### Cell viability assay

Aliquots of a TE8 cell suspension were mixed with trypan blue dye and left for 5 min at room temperature. Cells were counted using a hemocytometer and the percentage of live cells was determined by dye exclusion.

### Migration and invasion assay

*In vitro* Matrigel (Becton Dickinson, NJ, USA) invasion assays were done using 6.5 mm Costar transwell chambers (Costar Inc., CA, USA). The Transwell filters were coated with Matrigel, and cells were seeded onto the Matrigel. After incubation, the filter was removed from the chamber, and cells that had invaded the Matrigel were fixed and stained with hematoxylin and eosin. The number of cells attached to the filter was counted under a light microscope. The migration assay was conducted in a similar manner as the invasion assay, except that filters were not coated with Matrigel. Assays were repeated at least 3 times.

### Immunocytochemistry

TE8 cells were washed and fixed with 4% formaldehyde in PBS, and permeabilized using 0.2% NP-40 in PBS. Cells were blocked with bovine serum albumin for 1 hr, and then incubated with anti-paxillin (BD Biosciences) and an anti-mouse rhodamine-conjugated secondary antibody (Santa Cruz Biotechnology) or Alexa 488 phalloidin (Invitrogen) containing 1 mg/ml Hoechst (Invitrogen). Following incubation slides were mounted with anti-fading mounting medium (Dako North America). Cells were visualized and images were collected using fluorescence microscopy (Axiovert, Zeiss, Germany).

### Lactate and ATP assay

Lactate levels and ATP production in cell lysates were measured by a colorimetric L-Lactate assay kit (Abcam) and EnzyLight^TM^ ADP/ATP Ratio Assay kit (BioAssay Systems) according to the manufacturer's instructions. The total number of cells was used for normalization.

### Glucose uptake assay and flow cytometry

TE8 cells were plated at 2 × 10^5^/well in 6-well plates and incubated for 24 hr. Cells were then transfected with non-silencing siRNA or V1E1 siRNA for 72 hr using G-fectin (GP-2000). Culture medium was removed from each well and replaced with 2 ml culture medium in the presence or absence of 10 μM fluorescent 2-deoxy-2-[(7-nitro-2,1,3- benzoxadiazol-4-yl)amino]-D-glucose (2-NBDG). Cells were incubated at 37°C in 5% CO2 for 1 hr before flow cytometric analysis. Uptake of 2-NBDG was stopped by removing the incubation medium and washing the cells twice with pre-chilled phosphate buffered saline (PBS). Cells in each well were subsequently resuspended in 500 μl pre-chilled fresh growth medium and then stained with Propidium Iodide (PI) (1 μg/ml) and maintained at 4°C. Flow cytometric analysis was performed within 30 min using a BD FACS Canto II (San Jose, CA, USA).

### Glycolysis and OXPHOS measurements

Glycolysis and Oxidative phosphorylation (OXPHOS) were measured as extracellular acidification rate (ECAR) and oxygen consumption rate (OCR), respectively, using Seahorse XF24 extracellular flux analyzers according to the manufacturer's instructions. ECAR and OCR analysis was performed using the seahorse XF24 software (Version 1.7.0.74).

### TCGA and GSEA

Normalized gene expression data for ESCC tumors (RNA sequencing) were obtained for 1176 ESCC from The Cancer Genome Atlas. Gene set enrichment analysis (GSEA) software was used to perform GSEA against the TCGA database. Correlations between V-ATPase V1E1 and PKM2 mRNA expression levels in ESCC were determine from data generated by the TCGA Research Network (Available online: http://cancergenome.nih.gov/).

### Statistical analysis

The association between disease-specific survival and protein expression status was determined using the Kaplan-Meier method, and was compared using the log-rank test. Cox proportional hazards models were fitted for multivariate analysis to determine the prognostic effect of protein expression and clinicopathologic factors. Results on viability, migration, and invasion were analyzed by one-way, two-way analysis of variance (ANOVA), or Student's *t* test. Significant differences between means were determined using Bonferroni multiple comparisons test. Statistical analysis was performed using SPSS 17.0 for Windows (SPSS, Inc, Chicago, IL). All assay data are presented as mean ± SEM. A two-sided significance level of 0.05 was used for all statistical analyses.

## SUPPLEMENTARY MATERIALS FIGURES AND TABLES


