# Supplementary material for: Prognostic significance and function of the vacuolar H+-ATPase subunit V1E1 in esophageal squamous cell carcinoma
Source: Oncotarget. 2016 Jun 30;7(31):49334–48. doi: 10.18632/oncotarget.10340 (PMC5226512; doi:10.18632/oncotarget.10340)
Supplement: Supplementary file 1 [file oncotarget-07-49334-s001.pdf]

Prognostic significance and function of the vacuolar H^+-ATPase subunit V1E1 in esophageal squamous cell carcinoma

Supplementary Materials

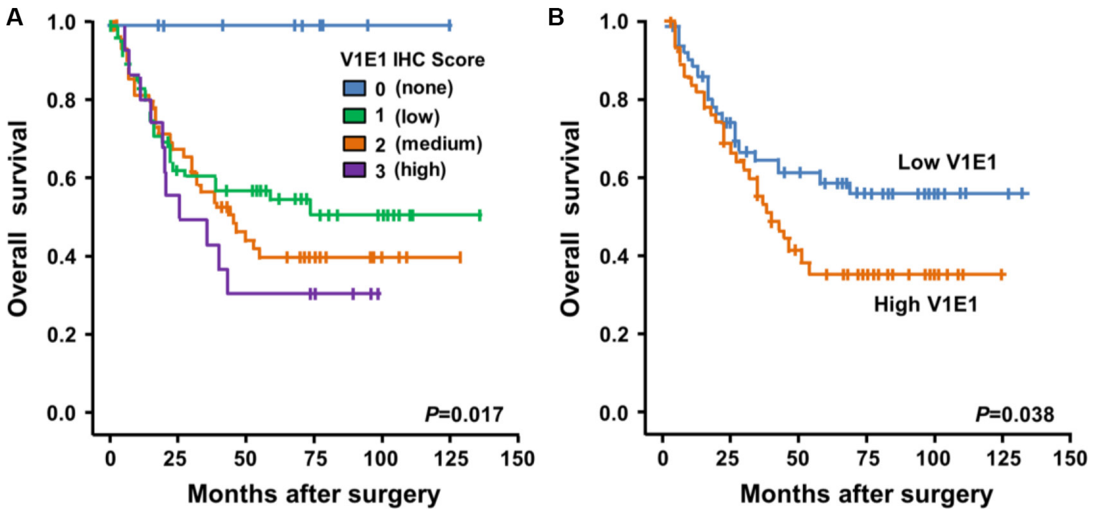

Supplementary Figure S1: Kaplan-Meier survival curves for overall survival according to the results of V-ATPase V1E1 immunostaining. (**A**) Kaplan-Meier curves showing overall survival among patients with ESCC on the basis of V-ATPase V1E1 expression status; (IHC score = 0, $n = 12$; IHC score = 1, $n = 72$; IHC score = 2, $n = 60$; IHC score = 3, $n = 16$). (**B**) Overall survival among patients with ESCC on the basis of V-ATPase V1E1 expression status; low V-ATPase V1E1 (IHC score < 2, $n = 84$) and high V-ATPase V1E1 (IHC score ≥ 2, $n = 76$). *P* values are from Log-Rank test.

Supplementary Table S1: Patients characteristics

Characteristics	Case no.(n = 302)	%
Age		
≥ 65 years	269	89
< 65 years	33	11
Sex		
Male	286	95
Female	16	5
Tumor size		
≥ 4.0 cm	110	36
< 4.0 cm	192	64
Differentiation		
W/D	61	21
M/D	185	61
P/D	56	18
TNM Stage		
I	55	18
II	87	29
III	109	36
IV	51	17
Tumor invasion		
T1	61	20
T2	52	17
T3	181	60
T4	8	3
LN metastasis		
Negative	120	40
Positive	182	60
Distant metastasis		
Absent	249	82
Positive	53	18

Supplementary Table S2: Primers used for quantitative PCR

Primer name	sequence
**hV-ATPase V1E1 F**	5′-GCA CAA GCC GAC CTT TCT-3′
**hV-ATPase V1E1 R**	5′-TTC ACC GGC CTA GCA TTG -3′
**hHIF-1a F**	5′-CGT TCC TTC GAT CAG TTG TC-3′
**hHIF-1a R**	5′-TCA GTG GTG GCA GTG GTA GT-3′
**hGlut1-F**	5′-CGG GCC AAG AGT GTG CTA AA-3′
**hGlut1-R**	5′-TGA CGA TAC CGG AGC CAA TG-3′
**hHK1-F**	5′-TGG AGT CCG AGG TTT ATG-3′
**hHK1-R**	5′-TTT GGA TTG TTG GCA AGG-3′
**hPFK F**	5′-CAT GAC CCA TGA AGA GCA CC-3′
**hPFK R**	5′-CCA ACT CGA ACC ACA GCC CTG-3′
**hENO1 F**	5′-CGT ACC GCT TCC TTA GAA C-3′
**hENO1 R**	5′-CAA TGA CTT GGG CCA ATT AC-3′
**hPKM2-F**	5′-GCC TGC TGT GTC GGA GAA G-3′
**hPKM2-R**	5′-CTC TCC CAG GAC CTT CCT AA-3′
**hLDHA-F**	5′-ATC TTG ACC TAC GTG GCT TGG A-3′
**hLDHA-R**	5′-CCA TAC AGG CAC ACT GGA ATC TC-3′
**hVimentin F**	5′-GGG ACC TCT ACG AGG AGG AG-3′
**hVimentin R**	5′-CGC ATT GTC AAC ATC CTG TC-3′
**hMMP2 F**	5′-CCA CTG CCT TCG ATA CAC-3′
**hMMP2 R**	5′-GAG CCA CTC TCT GGA ATC TTA AA-3′
**hMMP7 F**	5′-GAG TGA GCT ACA GTG GGA ACA-3′
**hMMP7 R**	5′-CTA TGA CGC GGG AGT TTA ACA T-3′
**h18s rRNA F**	5′-CGG CGA CGA CCC ATT CGA AC-3′
**h18s rRNA R**	5′-GAA TCG AAC CCT GAT TCC CCG TC-3′
